# Oxidation Behavior of TiCN-HfC-WC Cermet at High Temperature

**DOI:** 10.3390/ma19122648

**Published:** 2026-06-19

**Authors:** Zhihui Wang, Jiaojiao Gao, Jiabao Liu, Jinpeng Song

**Affiliations:** 1 College of Mechanical Engineering, Taiyuan University of Technology, Taiyuan 030024, Chinaliujiabao199802@163.com (J.L.); songjinpeng@tyut.edu.cn (J.S.); 2 College of Aeronautics and Astronautics, Taiyuan University of Technology, Taiyuan 030024, China

**Keywords:** TiCN-HfC-WC cermet, oxidation behavior, thermal fracture, high temperature

## Abstract

In this investigation, with increasing oxidation time or temperature, the observed mass gain abided by the parabolic law. TiCN-HfC-WC cermet contained different layers, with each one having a unique composition. Thermal fracture occurred in the sub-oxidation layer, and the flexural strength gradually decreased from 1270.6 MPa to 149.9 MPa. Ther-mal stress equations for calculating the radial, circumferential, and axial stresses were es-tablished. The thermal stress differences between neighboring layers determined the ther-mal fracture; when they were zero, the physical parameters of the layers were related, which could be used to guide cermet material design.

## 1. Introduction

TiCN-based cermet has good physical and chemical properties, such as high hardness, high resistance to deformation, high flexural strength, and high fracture toughness [1,2,3]. It has been widely applied to manufacture cutting tools [4,5,6]. In traditional machining, TiCN-based cermet tools exhibit good cutting performance and a long service life. With the development of high-speed dry cutting technology, TiCN-based cermet tools have faced severe challenges. In high-speed dry machining, the temperature between the cutting edge and workpiece, which is affected by friction heat, is approximately between 800 °C and 1100 °C, resulting in high requirements for TiCN-based cermet tools. In particular, TiCN-based cermet materials must maintain good oxidation resistance at high temperatures because this property is directly related to the degradation level and service life of TiCN-based cermet tools.

In recent years, many scholars worldwide have investigated the high-temperature oxidation behavior of several TiCN-based cermets. It was found that the mass gain of TiCN was small at low oxidation temperatures below 500 °C but large at high oxidation temperatures above 700 °C [7]. At 750 °C, the mass gain of Ti(C,N)-TaC-NbC abided by the parabolic rule, and the high temperature accelerated oxygen diffusion into the cermet inner layer [8]. At 900 °C, slight oxidation occurred in Al_2_O_3_/Ti(C,N) ceramic, and the oxide TiO_2_ prevented further oxidation [9]. At 900 °C, Ti(C,N)-AlN cermet suffered severe oxidation, and the main oxide was TiO_2_ [10]. At 900 °C, both the TiN and TiCN layers in multilayer TiCN/Al_2_O_3_/TiN coatings were oxidized to a porous TiO_2_ scale [11]. At 1000 °C, severe oxidation occurred in TiCN-WC cermet, and the major oxides were TiO_2_, WO_3_, N_2_, and CO/CO_2_ [12]. At 800–1100 °C, the oxide layers of Ti(C,N)-WC-Cr_3_C_2_ cermet consisted of a NiO outer layer, a NiTiO_3_ interlayer, and a TiO_2_-based inner layer [13]. At 800–1100 °C, Ti(C,N)-AlCoCrFeNi cermet exhibited superior oxidation resistance during the early oxidation stages because the dense outer oxide scale impeded the release of gaseous WO_3_ and inward oxygen diffusion [14]. When Ta was added to Ti(C,N)-based cermet, the formed Ti_0.95_Ta_0.05_C_0.5_N_0.5_ improved its oxidation resistance [15]; moreover, when a higher amount of Ta was added, the cermet showed outstanding oxidation resistance [16]. With increasing ZrC content, the oxidation resistance of Ti(C,N)-ZrC cermet gradually improved, and its mass gain followed the parabolic law in the first 60 h [17]. When an appropriate amount of Zr was added to Ti(C,N)-based cermet, at high temperatures, a dense layer formed on its surface, which reduced oxygen diffusion and improved its oxidation resistance [18]. To summarize, these studies mainly focused on the high-temperature oxidation behavior of TiCN-based cermet but failed to investigate its thermal fracture mechanism. The thermal fracture mechanism can play a vital role in understanding the formation of multilayer structures and thermal microcracks in the inner layers of oxidized cermet. The thermal fracture mechanism can be identified by analyzing the thermal stress in the material inner layers [19,20,21].

In summary, the effects of both the thermal stress-induced thermal fracture phenomenon during the oxidation of TiCN-based cermets and the regulatory mechanism of HfC incorporation on their oxidation behavior and thermal fracture characteristics remain to be systematically revealed. In this study, TiCN-HfC-WC cermet with a limited metal content was employed, its high-temperature oxidation behavior was investigated, and a thermal fracture model was established.

## 2. Experimental Procedures

**Figure 1:**
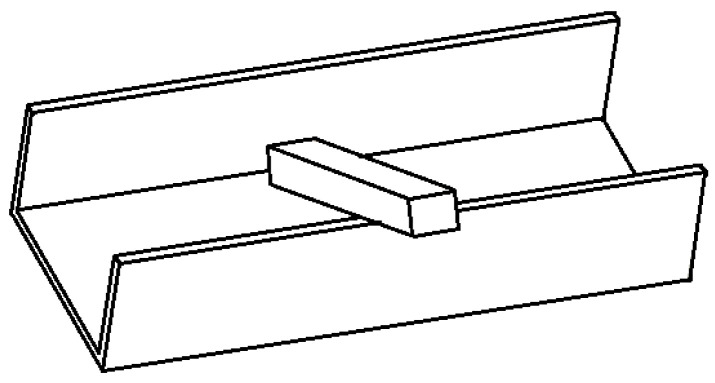
Placement of the TiCN-HfC-WC cermet specimen in the alumina crucible.

TiCN-HfC-WC cermet (THW), the fabrication, microstructure, and mechanical properties of which are reported in [22], contained 40 wt.% TiC_0.7_N_0.3_, 20 wt.% HfC, 32 wt.% WC, 4 wt.% Ni, and 4 wt.% Mo, and its flexural strength was 1270.6 MPa. The dimensions of the THW specimens were 3 mm × 4 mm × 40 mm, and their surface roughness was approximately 0.93 μm. The THW specimens were placed in an alumina crucible, as shown in Figure 1, to reduce their contact, and then they were placed in a muffle furnace (ZDXS5-2.5-120, Shenzhen Zhongda Strong Electric Furnace Co., Ltd., Shenzhen, China) under static lab air conditions. As detailed in Table 1, the THW specimens were repeatedly oxidized at different temperatures for different times. To determine the mass gain of each THW specimen, the mass of the tested specimen was measured before and after oxidation. After oxidation, the flexural strength was determined using three-point bending tests, which were carried out according to the standard GB/T 6569-2006/ISO 14704:2000 [23,24]. The tests were conducted on an electronic universal testing machine (GREE-8003G, Dongguan Kairui Technology Co., Ltd., Dongguan, China), with a span of 30 mm and a crosshead speed of 0.5 mm/min. The phase composition of the oxidized TiCN-HfC-WC cermet specimens was determined via X-ray diffraction (XRD, TD3600, Dandong Tongda Technology Co., Ltd., Dandong, China). The XRD tests were performed with Cu Kα radiation at a wavelength of 0.154 nm, a tube voltage of 40 kV, a tube current of 30 mA, and a 2θ scanning range of 20–80°. The surface morphology and fracture microstructure of the polished specimens were observed using a scanning electron microscope (SEM, JSM-7001F, JEOL Ltd., Tokyo, Japan). Energy-dispersive spectrometry (EDS, QUANTAX, Bruker, Karlsruhe, Germany) was used to analyze the chemical composition of the samples.

In addition to the XRD pattern collected from the specimen oxidized at 800 °C for 10 h, XRD patterns were also obtained from specimens oxidized for 10 h at 900, 1000, and 1100 °C in order to evaluate the temperature-dependent phase evolution of the oxidation products. The diffraction data were used for qualitative phase identification and quantitative phase analysis via Rietveld refinement using GSAS-II. The refined phase fractions are reported as relative weight fractions of the detected crystalline phases.

## 3. Results and Discussions

### 3.1. Oxides in and Mass Gain of TiCN-HfC-WC Cermet After Oxidation

#### 3.1.1. Oxides in TiCN-HfC-WC Cermet After Oxidation

**Figure 2:**
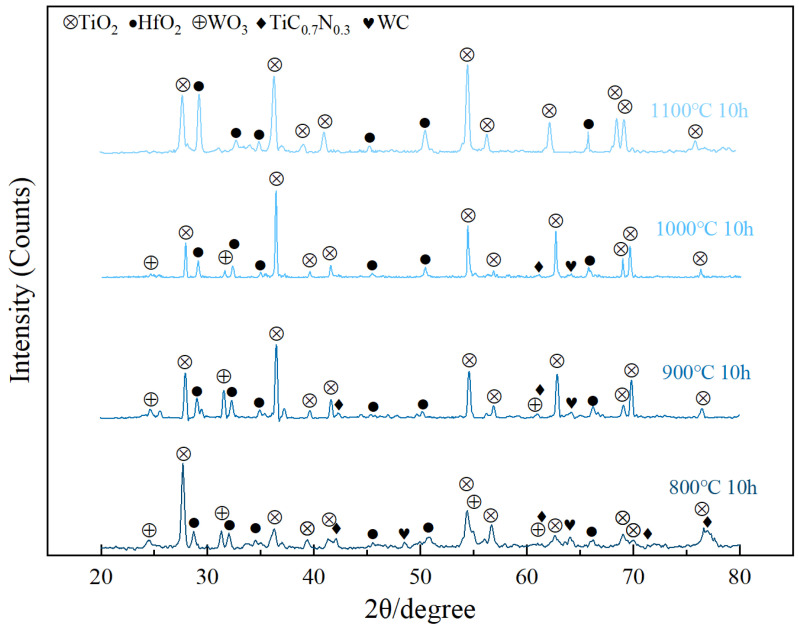
XRD of TiCN-HfC-WC cermet after oxidation at 800, 900, 1000, and 1100 °C for 10 h.

Figure 2 presents the XRD patterns of THW after oxidation for 10 h at 800, 900, 1000, and 1100 °C. At 800 °C, the major oxidation products were TiO_2_, WO_3_, and HfO_2_, while weak reflections of TiC_0.7_N_0.3_ and WC were still detected, indicating incomplete oxidation of the near-surface region. At 900 °C, TiO_2_ and HfO_2_ reflections became stronger, WO_3_ was still clearly present, and the residual TiC_0.7_N_0.3_/WC peaks remained weak but detectable. At 1000 °C, the diffraction pattern was dominated by TiO_2_ and HfO_2_, whereas WO_3_ became only a trace phase, and the residual carbide/carbonitride peaks were negligible. At 1100 °C, corresponding to the final oxidation stage investigated in this work, the retained oxide scale was mainly composed of TiO_2_ and HfO_2_, while tungsten-containing oxide was no longer a major crystalline phase and only trace minor oxides were detected. These results demonstrate progressive oxidation of TiC_0.7_N_0.3_, HfC, and WC with increasing temperature, together with strong depletion of WO_3_ from the retained oxide scale at high temperatures. Moreover, a previous study [25] reported that NiO, NiTO_3_, and NiMoO_4_ were present in the oxides after the oxidation of Ti(C,N)-based cermet, with a total content about 48 wt.% of Ni and Mo. The total content of Ni and Mo was much higher than 8 wt.% in this investigation, which accounted for these Ni-based oxides. In this investigation, it was difficult to exclude the existence of a small amount of these Ni-based oxides, although the total content of Ni and Mo was lower. According to thermodynamic calculations, the following oxidizing reactions accounted for the formation of these oxides:

TiC_0.7_N_0.3_(s) + 1.7O_2_(g) → TiO_2_(s) + 0.7CO_2_(g) +0.15N_2_(g) (1)

HfC(s) + 2O_2_(g) → HfO_2_(s) + CO_2_(g) (2)

WC(s) + 2.5O_2_(g) → WO_3_(s) + CO_2_(g) (3)

Quantitative phase analysis via Rietveld refinement was conducted to further clarify the oxidation-induced phase evolution. The TiO_2_ fraction increased from approximately 53.6 wt.% at 800 °C to 59.8 wt.% at 900 °C, 65.3 wt.% at 1000 °C, and 68.9 wt.% at 1100 °C. HfO_2_ increased from 16.2 wt.% at 800 °C to 18.5 wt.% at 900 °C and 33.0 wt.% at 1000 °C, followed by a slight decrease to 29.2 wt.% at 1100 °C. In contrast, WO_3_ decreased continuously from 24.1 wt.% at 800 °C to 17.7 wt.% at 900 °C and only 2.1 wt.% at 1000 °C, and it was no longer a major retained oxide phase at 1100 °C. Residual unoxidized phases accounted for about 5 wt.% at 800–900 °C but became negligible at 1000–1100 °C. Therefore, the oxidation scale evolved from a TiO_2_/WO_3_/HfO_2_ mixture with residual matrix phases at lower temperatures to a TiO_2_/HfO_2_-rich scale at higher temperatures.

Based on reaction (1), regardless of the generated CO_2_ and N_2_ gases, it is known that one mole of TiC_0.7_N_0.3_ could generate one mole of solid TiO_2_. Because the molar mass of TiO_2_ was larger than that of TiC_0.7_N_0.3_, the generated TiO_2_ had a larger mass than its counterpart TiC_0.7_N_0.3_; therefore, reaction (1) was classified as a mass gain reaction. Likewise, reactions (2) and (3) were also classified as mass gain reactions. Taken together, these results indicate that the mass of THW increased after oxidation. Meanwhile, the oxides TiO_2_, HfO_2_, and WO_3_ had a lower density than their counterparts TiC_0.7_N_0.3_, HfC, and WC, respectively; accordingly, the volume of THW expanded after oxidation. Although WO_3_ partially volatilized at high temperatures, the total mass gain still increased because the formation of TiO_2_ and HfO_2_ dominated the overall oxygen uptake during oxidation.

**Figure 3:**
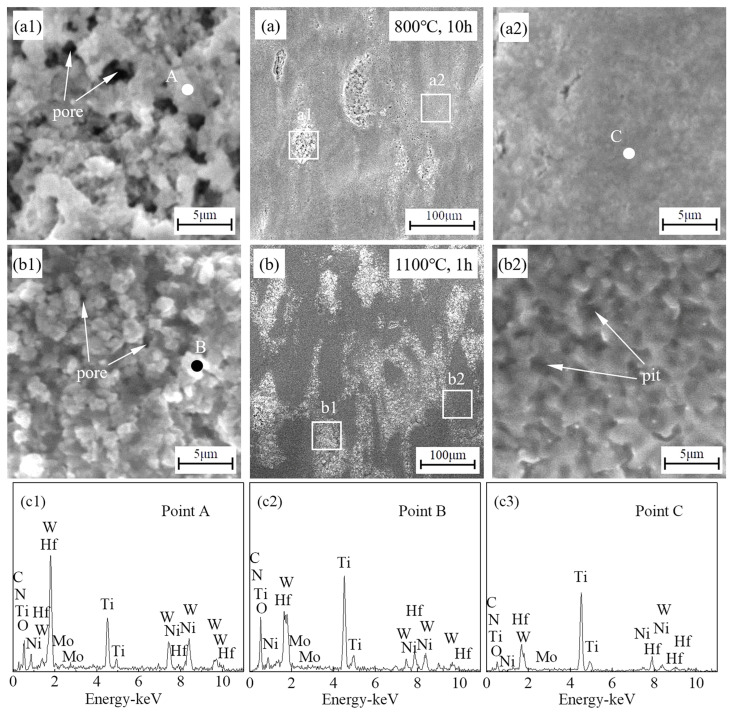
Polished surface morphologies and EDS of TiCN-HfC-WC cermet after oxidation: (**a**) the sample oxidized at 800 °C for 10 h; (**b**) the sample oxidized at 1100 °C for 1 h; (**c**) EDS point scanning results of points A, B and C.

Figure 3 shows the polished surface morphologies of THW after oxidation. As can be seen in Figure 3a,b, several areas (rectangles a1 and b1) were filled with white particles, named white particle areas. During the oxidation process, the residual pores or microcracks on the surface of the original cermet provided channels for oxygen to enter the inner layer and thus further oxidize the material; they also provided escape paths for the produced CO_2_ and N_2_ gases, which could explain the formation of the surface morphologies in rectangles a1 and b1 in Figure 3a,b, respectively. Moreover, a higher number of these areas was observed in Figure 3b than in Figure 3a, which indicates that a high temperature of 1100 °C could lead to more residual pores or microcracks, thereby promoting the oxidation process.

To determine the composition of the oxidation surface, larger views and EDS were employed. Figure 3(a1,a2) represent the two different areas in Figure 3a, and Figure 3(b1,b2) represent the two different areas in Figure 3b. Figure 3(c1–c3) present the EDS of Points A, B, and C, respectively. Moreover, Table 2 details the element contents of the corresponding points. According to the XRD, EDS, and element content results, it was inferred that the main components of the cermet after oxidation were TiO_2_ and WO_3_ at Point A; TiO_2_, HfO_2_, and WO_3_ at Point B; and TiCN, TiO_2_, HfO_2_, and WO_3_ at Point C. As detailed in Table 3, the W atomic percentage of 2.2% at Point B is less than the 9.1% at Point A, which likely resulted from the volatilization of WO_3_ above 850 °C [12,13].

In addition, many pores can be observed in Figure 3(a1), and they are larger than those in Figure 3(b1). During the oxidation process, at 800 °C, when the oxidation time increased from 0 to 10 h, some pores or microcracks were compressed and disappeared as a result of the expansion and growth of TiO_2_, HfO_2_, and WO_3_; however, some still provided channels for gas escape, thereby leading to the formation of large pores. At 1100 °C, a large number of the original pores and microcracks were inclined and rapidly compressed until they disappeared due to the expansion and growth of TiO_2_ or HfO_2_, owing to the shorter oxidation time; if the oxidation time were longer, many large pores may have been observed in Figure 3(b1). Moreover, many pits can be observed in Figure 3(b2), but almost none can be observed in Figure 3(a2). Pit formation could be attributed to the volatilization of WO_3_.

#### 3.1.2. Mass Gain of TiCN-HfC-WC Cermet After Oxidation

**Figure 4:**
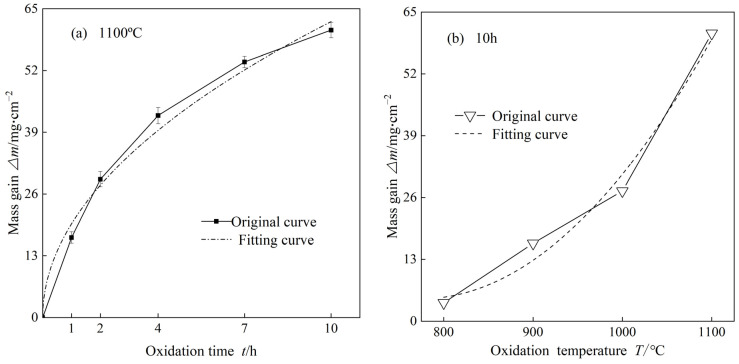
Mass gain of TiCN-HfC-WC cermet with oxidation time and temperature: (**a**) oxidation time; (**b**) oxidation temperature.

Figure 4a shows the mass gain of THW as a function of the oxidation time at 1100 °C. Based on the original curve, it was found that, after increasing the oxidation time from 0 to 10 h, the mass gain increased gradually from 0 to 60.5 mg/cm^2^. Based on the original curve, a fitting curve was drawn, as shown in Figure 4a, and its equation was as follows:

(4) $$\Delta m^{2}=387.69t$$

where Δ*m* is the mass gain, and *t* is the oxidation time. Based on Equation (4), it was found that the fitting curve was a parabolic curve and that the original curve approached it; therefore, it was determined that the oxidation of THW followed the parabolic law.

Figure 4b shows the mass gain of THW as a function of the oxidation temperature for 10 h. Based on the original curve, it was found that, after increasing the oxidation temperature from 800 °C to 1100 °C, the mass gain increased gradually from 3.83 to 60.5 mg/cm^2^, indicating that the THW cermet maintained better oxidation resistance at 800 °C. Based on the original curve, a fitting curve was drawn, as shown in Figure 4b, and its equation was as follows:

(5) $$\Delta m=5.16\times 10^{-4}T^{2}-0.79T+314.27$$

where Δ*m* is the mass gain, and *T* is the oxidation temperature. Equation (5) is a quadratic equation, which explains the relationship between the mass gain and oxidation temperature in this investigation.

### 3.2. Oxidation Mechanism of TiCN-HfC-WC Cermet

**Figure 5:**
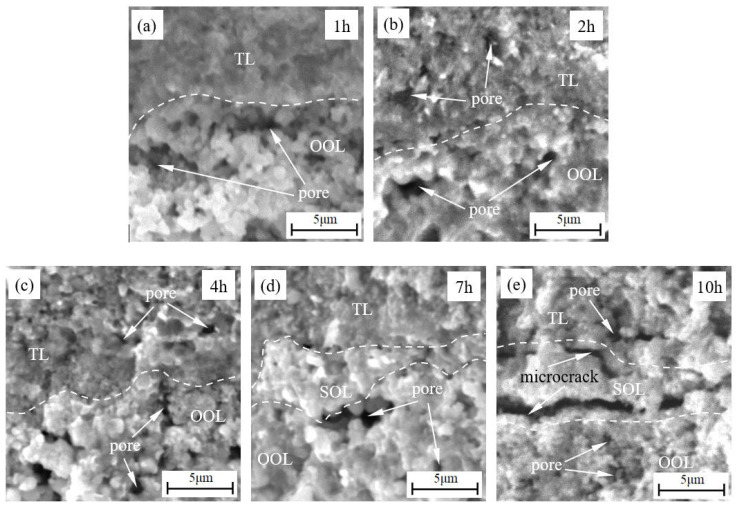
Fracture surface morphologies of TiCN-HfC-WC cermet after oxidation at 1100 °C: (**a**) oxidized for 1 h; (**b**) 2h; (**c**) 4h; (**d**) 7h; (**e**) 10h.

Figure 5 shows the fracture surface morphologies of THW after oxidation at 1100 °C for 1 h, 2 h, 4 h, 7 h, and 10 h. After increasing the oxidation time from 1 h to 10 h, the oxidation of the cermet became more severe. Generally, oxidation characteristics were observed in the layers, except for in the substrate layer [16,17,18]. To obtain more details of these oxidation characteristics, the layers, except for the substrate layer, became the main focus of this study. According to the morphologies of the oxidized cermets, their fracture surfaces were divided into different segments using white dashed curves. The upper segment was located in the inner layer of the cermet and was named the transition layer (TL); the lower segment was located in the outer layer of the cermet, exhibited severe oxidation, and was named the outer oxidation layer (OOL); and the middle segment was located between the upper and lower layers and was named the sub-oxidation layer (SOL).

As can be seen in Figure 5a–e, after increasing the oxidation time from 0 to 10 h, the pore size first increased and then decreased in the OOL. As can be seen in Figure 5b,e, the TL exhibits several large pores, but in Figure 5c, it exhibits several small pores. When the oxidation time increased from 0 to 1 h, the outer surface of the cermet made direct contact with oxygen, thereby leading to the rapid formation of oxides, and the formed gases (CO_2_ and N_2_) and volatile matter (WO_3_) escaped from the outer surface. The residual pores and microcracks on the surface also provided channels for oxygen to enter and erode the inner cermet layer, leading to the production of gases and a certain amount of volatile matter. Because of the shorter oxidation time, the pores in the OOL were relatively small, and very slight oxidation occurred in the TL. When the oxidation time increased from 1 h to 2 h, the residual pores and microcracks, under the pressure of the gases and volatile matter in the inner cermet layers, became larger, which facilitated the invasion of oxygen and the release of these gases and volatile matter, leading to a further reduction in WO_3_ in the OOL. This also enabled oxygen to directly erode the cermet, leading to severe oxidation and large pores in the TL. When the oxidation time increased from 2 h to 4 h, a large amount of the formed HfO_2_ and TiO_2_ grew and expanded, thereby compressing the large pores to small pores in the OOL and TL. When the oxidation time increased from 4 h to 7 h, this compression continued, resulting in few large pores and small pores in the OOL and almost no pores in the TL. Thus, oxidation gradually entered a new stage, namely, diffusion oxidation, through the inward diffusion of O. Moreover, a new layer (SOL) formed between the OOL and TL, owing to the different compositions of these layers. The OOL contained a large amount of TiO_2_ and HfO_2_; the SOL contained a large amount of TiO_2_ and HfO_2_ with a small amount of WO_3_; and the TL contained a small amount of HfO_2_ and TiO_2_. This difference could probably be explained by the reaction priority of the materials under the same reaction conditions [26,27,28]. Regardless of the limited Ni and Mo contents in the cermet, according to the Gibbs free energies of TiC_0.7_N_0.3_, WC, and HfC, when they react with oxygen, their reaction priority is in the order (2), (1), and (3), as previously mentioned. Therefore, it is likely that the oxides formed in the order HfO_2_, TiO_2_, then WO_3_. The OOL contained a large amount of HfO_2_ and TiO_2_ because WO_3_ was easily volatilized. The SOL contained a large amount of HfO_2_ and TiO_2_ with a small amount of WO_3_ because the formation of WO_3_ was limited by the reaction priority and its volatilization was limited by pore closure. The TL contained a small amount of HfO_2_ and TiO_2_ because their formation was limited by the inward diffusion speed of O. Accordingly, the HfO_2_ and TiO_2_ contents gradually decreased from the outer to inner layers, while the WO_3_ content first increased and then decreased because of its volatilization. Furthermore, each layer had a new, unique composition. When the oxidation time increased from 7 h to 10 h, the pores gradually disappeared in the OOL, owing to the further growth and expansion of the oxides. Moreover, a fault zone formed in the SOL, which was caused by thermal stress and the pressure of the formed gases. The microcracks between the SOL and TL provided a channel for oxygen to enter and further erode the TL, which resulted in several large pores in this layer.

**Figure 6:**
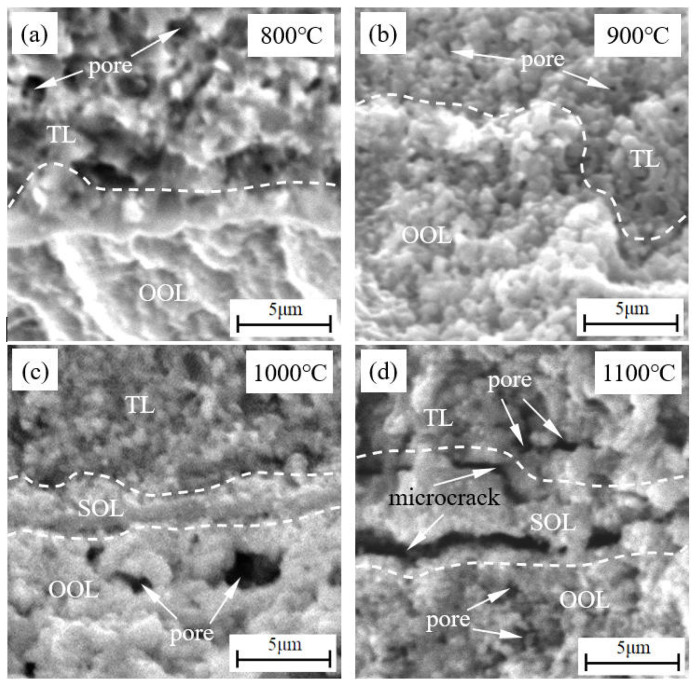
Fracture surface morphologies of TiCN-HfC-WC cermet after oxidation for 10 h: (**a**) oxidation temperature of 800 °C; (**b**) 900 °C; (**c**) 1000 °C; (**d**) 1100 °C.

Figure 6 shows the fracture surface morphologies of THW after oxidation for 10 h at different temperatures. With increasing oxidation temperature, THW demonstrated different microstructures. As shown in Figure 6a, after oxidation at 800 °C for 10 h, the OOL became relatively dense, and the main oxides were TiO_2_ and HfO_2_, with a small amount of WO_3_; furthermore, the TL exhibited many large pores. As shown in Figure 6b, after oxidation at 900 °C for 10 h, owing to the acceleration of oxygen diffusion by the temperature, a large amount of WO_3_ was produced in the OOL, and the pores in the TL gradually became small as a result of the growth and expansion of the oxides. As shown in Figure 6c, after oxidation at 1000 °C for 10 h, a large amount of WO_3_ volatilized, resulting in several large pores in the OOL; an SOL formed in the cermet because of the different compositions of the oxides; and slight oxidation occurred in the TL. As shown in Figure 6d, after oxidation at 1100 °C for 10 h, the OOL became dense, owing to the growth and expansion of the remaining oxides TiO_2_ and HfO_2_; the SOL developed a fault zone; and the TL developed microcracks, which promoted the progression of oxidation.

Combined with the fracture morphologies in Figure 6, the XRD/Rietveld results indicate that increasing the temperature gradually transformed the near-surface region into a TiO_2_/HfO_2_-rich oxide scale. WO_3_ was apparent at 800–900 °C but became only a trace constituent at 1000 °C and was no longer a major retained crystalline phase at 1100 °C, which is consistent with the volatilization-induced pits observed on the oxidized surface. Therefore, the outer oxidation layer at the highest temperatures was mainly controlled by TiO_2_ and HfO_2_, whereas tungsten-containing species contributed much less to the retained oxide scale.

### 3.3. Thermal Fracture of TiCN-HfC-WC Cermet in the High-Temperature Oxidation Process

**Figure 7:**
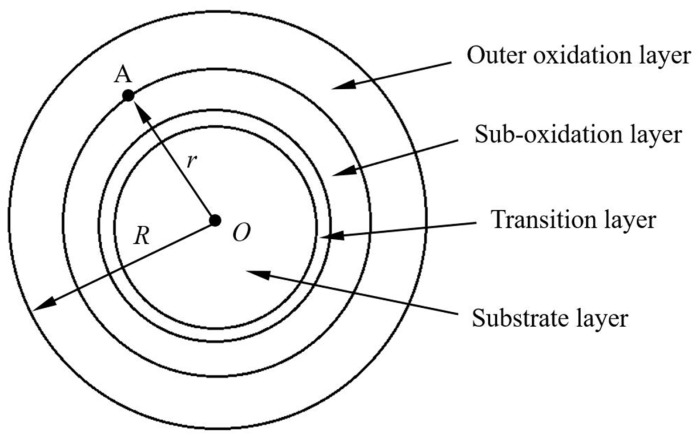
Layers in the TiCN-HfC-WC cermet after oxidation.

Regarding the layers on the fracture surfaces of THW, from the inner layer to the outer layer, the THW cermet (shown in Figure 7) was composed of a substrate layer, a transition layer, a sub-oxidation layer, and an outer oxidation layer. These layers, except for the substrate layer, were oxidation layers; their oxidation became more severe from the inner to the outer layer, and they exhibited new, unique compositions. Several microcracks developed between the SOL and OOL and between the SOL and TL, as previously mentioned, indicating that thermal fracture occurred in the cermet.

To identify the thermal fracture of this cermet in the oxidizing process, a homogeneous isotropic thermoelastic solid cylinder was employed to replace the cermet sample and analyze the effects of thermal stress on the layers. The assumption was as follows: the solid cylinder was of infinite length; its radius, length, and outer surface temperature were *R*, *L*, and *T*_0_, respectively; the outer heat source generated heat *W* per unit time and per unit volume to the solid cylinder; and the temperature *T* was a function of the radius *r* in a steady-state temperature field. Therefore, in cylindrical coordinates, the heat diffusion equation for a homogeneous solid cylinder can be expressed as follows:

(6) $$\frac{\partial T}{\partial t}-a\times (\frac{\partial^{2}T}{\partial r^{2}}+\frac{1}{r}\frac{\partial T}{\partial r}+\frac{1}{\partial r}\frac{\partial^{2}T}{\partial \theta^{2}}+\frac{\partial^{2}T}{\partial z^{2}})=\frac{W}{c\rho }$$

where *t* is the time, *a* is the temperature coefficient, *θ* is the circumferential coordinate, *z* is the axial coordinate, *c* is the specific heat capacity, and *ρ* is the density of the cermet.

Because the temperature field was in a steady state, $\frac{\partial T}{\partial t}=0$, $\frac{\partial T}{\partial \theta }=0$, and $\frac{\partial T}{\partial z}=0$. Moreover, the thermal conductivity λ=acρ. Hence, Equation (6) can be re-expressed as follows:

(7) $$\frac{d}{dr}(r\frac{dT}{dr})=-\frac{Wr}{\lambda }$$

By integrating Equation (7), the following equation is obtained:

(8) $$\frac{dT}{dr}=-\frac{W}{2\lambda }r+\frac{C_{1}}{r}$$

By introducing the boundary condition ${\left.-\lambda 2\pi RL\frac{dT}{dr}\right|}_{R}=W\pi R^{2}L$ into Equation (8), *C*_1_ = 0 can be obtained. Thus, Equation (9) can be expressed as follows:

(9) $$\frac{dT}{dr}=-\frac{W}{2\lambda }r$$

By integrating Equation (9), the following equation can be obtained:

(10) $$T=-\frac{W}{4\lambda }r^{2}+C_{2}$$

Then, by introducing the initial condition—*r* = *R*, *T* = *T*_0_—into Equation (10), $C_{2}=T_{0}+\frac{W}{4\lambda }R^{2}$ can be obtained. Thus, Equation (10) can be re-expressed as follows:

(11) $$T=T_{0}+\frac{W}{4\lambda }(R^{2}-r^{2})$$

To determine the thermal stress of a point in the THW cermet, Equation (11) is introduced into the following thermal stress equations:

(12) $$\sigma_{r}=\frac{\alpha E}{1-\nu }\left\{\frac{1}{R^{2}}\int_{0}^{R}Trdr-\frac{1}{r^{2}}\int_{0}^{r}Trdr\right\}$$

(13) $$\sigma_{\theta }=\frac{\alpha E}{1-\nu }\left\{\frac{1}{R^{2}}\int_{0}^{R}Trdr+\frac{1}{r^{2}}\int_{0}^{r}Trdr-T\right\}$$

(14) $$\sigma_{z}=\frac{\alpha E}{1-\nu }\left\{\frac{2}{R^{2}}\int_{0}^{R}Trdr-T\right\}$$

After integrating these equations, thermal stress equations are obtained as follows:

(15) $$\sigma_{r}=\frac{\alpha EW(r^{2}-R^{2})}{16\lambda (1-\nu )}$$

(16) $$\sigma_{\theta }=\frac{\alpha EW(3r^{2}-R^{2})}{16\lambda (1-\nu )}$$

(17) $$\sigma_{z}=\frac{\alpha EW(2r^{2}-R^{2})}{8\lambda (1-\nu )}$$

where $\sigma_{r}$, $\sigma_{\theta }$, $\sigma_{z}$, *α*, *E*, and *ν* are the radial stress, circumferential stress, axial stress, thermal expansion coefficient, Young’s elastic modulus, and Poisson’s ratio, respectively.

Equations (15)–(17) can be used to evaluate the radial, circumferential, and axial stresses at every point in the THW cermet, especially at points located at the interface between different neighboring layers. As previously mentioned, each layer had a different composition, which resulted in different physical and chemical properties. For example, Point A in Figure 7 was located at the interface between the outer oxidation and sub-oxidation layers. The interface divided Point A into two parts: one part was located in the outer oxidation layer, and the other was located in the sub-oxidation layer. Therefore, Point A had two sets of radial, circumferential, and axial stress values: one set was from the outer oxidation layer, and the other set was from the sub-oxidation layer, owing to the different physical and chemical properties of these layers. The difference in each set of radial, circumferential, and axial stress values can be calculated. For the stress differences at Point A, their absolute values $\left|\Delta \sigma_{r}\right|$, $\left|\Delta \sigma_{\theta }\right|$, and $\left|\Delta \sigma_{z}\right|$ can be calculated according to Equations (15)–(17) as follows:

(18) $$\left|\Delta \sigma_{r}\right|=\left|\sigma_{r1}-\sigma_{r2}\right|=\frac{W(R^{2}-r^{2})}{16}\left|\frac{\alpha_{1}E_{1}}{\lambda_{1}(1-\nu_{1})}-\frac{\alpha_{2}E_{2}}{\lambda_{2}(1-\nu_{2})}\right|$$

(19) $$\left|\Delta \sigma_{\theta }\right|=\left|\sigma_{\theta 1}-\sigma_{\theta 2}\right|=\frac{W\left|3r^{2}-R^{2}\right|}{16}\left|\frac{\alpha_{1}E_{1}}{\lambda_{1}(1-\nu_{1})}-\frac{\alpha_{2}E_{2}}{\lambda_{2}(1-\nu_{2})}\right|$$

(20) $$\left|\Delta \sigma_{z}\right|=\left|\sigma_{z1}-\sigma_{z2}\right|=\frac{W\left|2r^{2}-R^{2}\right|}{8}\left|\frac{\alpha_{1}E_{1}}{\lambda_{1}(1-\nu_{1})}-\frac{\alpha_{2}E_{2}}{\lambda_{2}(1-\nu_{2})}\right|$$

where $\sigma_{r1}$, $\sigma_{\theta 1}$, and $\sigma_{z1}$ are the thermal stresses caused by the material in the outer oxidation layer, and *α*_1_, *E*_1_, $\lambda_{1}$, and *ν*_1_ are the thermal expansion coefficient, Young’s elastic modulus, thermal conductivity, and Poisson’s ratio of the material in the outer oxidation layer, respectively; $\sigma_{r2}$, $\sigma_{\theta 2}$, and $\sigma_{z2}$ are the thermal stresses caused by the material in the sub-oxidation layer, and *α*_2_, *E*_2_, $\lambda_{2}$, and *ν*_2_ are the thermal expansion coefficient, Young’s elastic modulus, thermal conductivity, and Poisson’s ratio of the material in the sub-oxidation layer, respectively.

The allowable stresses of the material at Point A are marked as $\left[\sigma_{Ar}\right]$, $\left[\sigma_{A\theta }\right]$, and $\left[\sigma_{Az}\right]$. When $\left|\Delta \sigma_{r}\right|>\left[\sigma_{Ar}\right]$, for the solid cylinder, radial displacement occurred at Point A, as well as at the other points located on the same cylindrical surface, resulting in interface fracture and the formation of interface microcracks between the outer oxidation and sub-oxidation layers. When $\left|\Delta \sigma_{\theta }\right|>\left[\sigma_{A\theta }\right]$, for the solid cylinder, torsional deformation occurred at Point A, as well as at the other points located on the same generatrix, leading to radial fracture and the formation of radial microcracks. When $\left|\Delta \sigma_{z}\right|>\left[\sigma_{Az}\right]$, for the solid cylinder, circumferential displacement occurred at Point A, as well as the other points located on the same circle, causing circumferential fracture and the formation of circumferential microcracks. Aside from the gaseous pressure, these mechanisms could well explain the formation of the microcracks and fault zone in Figure 5e and Figure 6d. These microcracks provided channels for oxygen, allowing it make contact with exposed and unoxidized grains and then oxidize them, thereby leading to the formation of oxides. They also enabled the volatile oxide WO_3_ and the produced gases, such as N_2_ and CO_2_, to escape from the cermet inner layers, which could have accelerated the oxidation and degradation of the TL, as observed in Figure 5e and Figure 6d.

Based on the stress difference Equations (16)–(18), it was found that the stress differences at certain points had similar relationships with the physical parameters *α*, *E*, λ, and *ν*. When the stress differences were zero, fractures did not occur, and the physical parameters of the neighboring layers showed the following relationship:

(21) $$\frac{\alpha_{1}E_{1}}{\lambda_{1}(1-\nu_{1})}=\frac{\alpha_{2}E_{2}}{\lambda_{2}(1-\nu_{2})}$$

Equation (21) provides a potential approach to avoid thermal fracture and inhibit the oxidation of the cermet material, as the physical parameters of the oxides that form in neighboring layers could be balanced at the stage of cermet material design.

### 3.4. Flexural Strength of TiCN-HfC-WC Cermet After Oxidation

**Figure 8:**
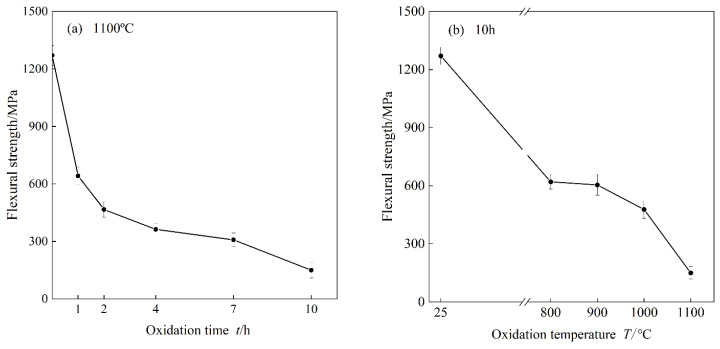
Flexural strength of TiCN-HfC-WC cermet after oxidation: (**a**) oxidation time; (**b**) oxidation temperature.

Figure 8 shows the flexural strength of THW after oxidation. As shown in Figure 8a, with increasing oxidation time from 0 to 10 h at 1100 °C, the flexural strength of the cermet gradually decreased from 1270.6 MPa to 149.9 Mpa. In particular, it sharply decreased by approximately 50% when the oxidation time increased from 0 to 1 h. The reason for this is as follows: a large amount of oxides was rapidly produced on the outer surface of the cermet, and, as a result, the whole outer surface was exposed to air; this allowed oxygen to make full contact with the cermet and oxidize it without any obstacles, thereby leading to a large decrease in the flexural strength. When the oxidation time increased from 1 h to 7 h, the decrease in the flexural strength slowed down, as the oxides protected the inner cermet layer from further oxidization. However, when the oxidation time increased from 7 h to 10 h, the flexural strength decreased sharply again; this was caused by the pores and microcracks, which not only reduced the mechanical properties of the cermet but also provided channels for oxygen to further oxidize it.

As shown in Figure 8b, with increasing oxidation temperature from 0 to 1100 °C, after 10 h, the flexural strength of the cermet gradually decreased from 1270.6 Mpa to 149.9 Mpa. In particular, it sharply decreased to 620.4 Mpa when the oxidation temperature increased from 0 to 800 °C. The reason for this is as follows: the high temperature provided a large amount of energy that promoted the oxidation of the whole outer cermet surface, and, as a result, a large amount of oxides was produced, leading to the rapid degradation of the cermet. From 800 °C to 900 °C, the decrease in the flexural strength slowed down, as the formed oxides prevented further oxidation. However, when the oxidation temperature increased from 900 °C to 1100 °C, the decrease in the flexural strength gradually accelerated, which was caused by an increase in oxides, pores, and microcracks in the cermet.

## 4. Conclusions

High-temperature oxidation behavior and thermal fracture of TiCN-HfC-WC cermet with limited metal content had been investigated. Some conclusions were as follows:

The high-temperature oxidation behavior and thermal fracture of TiCN-HfC-WC cermet with a limited metal content were investigated in this study. Several conclusions were drawn:

(1) After oxidation for 10 h at 800–1100 °C, the major oxidation products of the TiCN-HfC-WC cermet were TiO_2_, HfO_2_, and WO_3_. Qualitative XRD analysis showed residual TiC_0.7_N_0.3_/WC phases at 800–900 °C, whereas the oxide scale at 1000–1100 °C was dominated by TiO_2_ and HfO_2_. Rietveld refinement indicated that TiO_2_ increased from 53.6 wt.% at 800 °C to 68.9 wt.% at 1100 °C; HfO_2_ increased from 16.2 wt.% to 29.2 wt.%, reaching 33.0 wt.% at 1000 °C; and WO_3_ decreased from 24.1 wt.% to a trace amount, consistent with its volatilization at high temperature.
(2) After 1100 °C and 10 h oxidation, with increasing temperature and oxidation time, the cermet exhibited different microstructures: different layers formed, a fault zone appeared in the sub-oxidation layer, and the flexural strength gradually decreased from 1270.6 MPa to 149.9 MPa. Each layer had a new, unique composition because the HfO_2_ and TiO_2_ contents gradually decreased from the outer to inner layers, while the WO_3_ content first increased and then decreased. The quantitative phase evolution showed that the retained oxide scale became progressively enriched in TiO_2_ and HfO_2_ with increasing temperature, whereas WO_3_ progressively depleted.
(3) Thermal stress equations for calculating the radial, circumferential, and axial stresses were established. At specific oxidation temperatures, thermal stress differences between neighboring layers resulted in interface, radial, and circumferential fractures. When the thermal stress differences were zero, these fractures did not occur, and the physical parameters of neighboring layers, such as the thermal expansion coefficient, Young’s elastic modulus, thermal conductivity, and Poisson’s ratio, exhibited a certain relationship. This relationship provides a potential approach to avoid thermal fracture and inhibit the oxidation of cermet, as the physical parameters of the oxides that form in neighboring layers can be balanced during cermet material design.

## Figures and Tables

**Table 1:** Oxidation experiments of TiCN-HfC-WC cermet.

Test No.	1	2	3	4	5	6	7	8
Oxidation temperature/°C	1100	1100	1100	1100	1100	800	900	1000
Oxidation time/h	1	2	4	7	10	10	10	10

**Table 2:** Quantitative phase composition of TiCN-HfC-WC cermet after oxidation for 10 h at different temperatures, determined by Rietveld refinement of the XRD patterns.

Temperature	TiO_2_ Content	WO_3_ Content	HfO_2_ Content	Other Phase Content
800 °C, 10 h	53.6%	24.1%	16.2%	Residual phase ≈ 5%
900 °C, 10 h	59.8%	17.7%	18.5%	Residual phase ≈ 5%
1000 °C, 10 h	65.3%	2.1%	33.0%	—
1100 °C, 10 h	68.9%	—	29.2%	Ni-based oxides ≈ 2%

**Table 3:** Element content of points in Figure 3.

Element	Point A		Point B		Point C	
	wt./%	at./%	wt./%	at./%	wt./%	at./%
Ti K	17.3	14.1	39.4	28.5	48.4	38.7
C K	1.8	5.8	0	0	7.5	23.8
N K	0.7	1.9	0	0	2.9	7.8
O K	26.4	64.5	29.5	64.0	9.1	21.5
W L	43.1	9.1	11.6	2.2	17.6	3.7
Hf L	4.3	0.9	13.8	2.7	10.1	2.2
Ni K	4.0	2.7	2.7	1.6	2.3	1.5
Mo L	2.4	1.0	2.9	1.1	2.1	0.8
Total	100	100	100	100	100	100

## Data Availability

The original contributions presented in this study are included in the article. Further inquiries can be directed to the corresponding author.

## Abbreviations

The following abbreviations are used in this manuscript:

THW	TiCN-HfC-WC cermet
XRD	X-ray diffraction
SEM	scanning electron microscope
EDS	Energy dispersive spectrometry
TL	transition layer
OOL	outer oxidation layer
SOL	sub-oxidation layer
